# Hygienic Value of the Sun’s Rays

**Published:** 1899-04

**Authors:** 


### Hygienic Value of the Sun's Rays.

It is astonishing how few people there are who properly estimate the hygienic value of the suns' rays. A valuable lesson on this point may be learned by observing the lower animals, none of whom ever neglect an opportunity to bask in the sun. And the nearer man approaches to his primitive condition, the more he is inclined to follow the example of the animals. It is natural instinct, which civilization has partially destroyed in the human race.

The effect of the sunshine is not merely thermal, its rays have chemical and electric functions. It is more than possible that sunshine produces vibrations and changes of particles in the deeper tissues of the body as effective as those of electricity. Many know by experience that the relief it affords to wearing pain, neuralgic and inflammatory, is more effective and lasting than that of any application whatever. Those who have face ache should prove it for themselves, sitting in a sunny window, where the warmth falls full on the cheek. For nervous debility and insomnia the treatment of all others is rest in the sunshine. There is no tonic like it, provided the good effects are not neutralized by ill feeding. To restore a withered arm, a palsied or rheumatic limb, or to bring a case of nervous prostration up speedily, a most efficient part of the treatment would be to expose the limb or the person as many hours to direct sunlight as the day would afford. With weak lungs, let the sun fall full on the chest for hours. If internal humor or ulceration is suspected, let the sun burn through the bare skin, directly on the point of disease, for hours daily. There will be no doubt left in the mind that there is a curative power in the chemical rays of the sun. For the chilliness which causes blue hands and bad color, resort to the sun—let it almost blister the skin, and the circulation will answer the attraction. It is a finer stimulus than wine, electricity or massage, and we are on the verge of great therapeutic discoveries concerning it. —*Ex.*

